# (*R*)-2-[1-(2,6-Dichloro-3,4,5-trimeth­oxy­benzo­yl)pyrrolidin-2-yl]-4,4,5,5-tetra­methyl-4,5-dihydro-1*H*-imidazole-1-oxyl 3-oxide

**DOI:** 10.1107/S1600536811010270

**Published:** 2011-03-26

**Authors:** Hai-Bo Wang, Zhuo Xiang, Lin-Lin Jing, Min Tian, Xiao-Li Sun

**Affiliations:** a Department of Chemistry, School of Pharmacy, Fourth Military Medical University, Changle West Road 17, 710032 Xi-An, People’s Republic of China; b Department of Pharmacy, Lanzhou General Hospital, Lanzhou Command, Lanzhou 730050, People’s Republic of China

## Abstract

In the title compound, C_21_H_28_Cl_2_N_3_O_6_, the nitronyl nitroxide ring displays a half-chair conformation, whereas the pyrrolidine ring has an envelope conformation. These two rings are twisted to each other with N—C—C—N torsion angles around the connecting C—C bond of 48.9 (6) and −127.0 (5)°. The benzene ring is nearly perpendicular to the pyrrolidine ring, with torsion angles around the connecting C—C bond of 86.3 (6) and −97.7 (6)°. The crystal structure is stabilized by C—H⋯O and C—H⋯π hydrogen bonds, which build up a three-dimensional network.

## Related literature

For the chemical and physical properties of nitronyl nitroxides, see: Minguet *et al.* (2001 ▶); Osiecki & Ullman (1968 ▶); Shemsi *et al.* (2007 ▶); Wu *et al.* (2006 ▶). For related structures, see: Shimono *et al.* (2004 ▶); Minguet *et al.* (2001 ▶); Tian *et al.* (2011 ▶). For puckering parameters, see Cremer & Pople (1975 ▶). For a description of the Cambridge Structural Database, see: Allen (2002 ▶). 
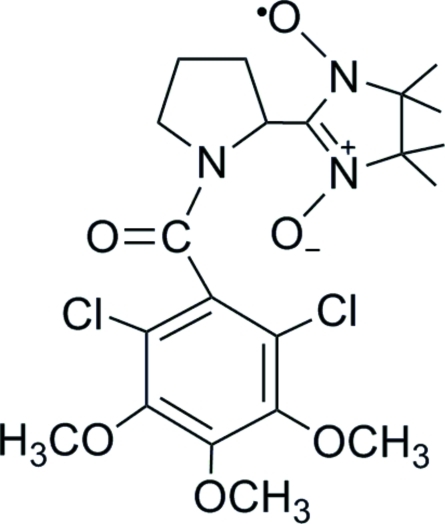


## Experimental

### 

#### Crystal data

C_21_H_28_Cl_2_N_3_O_6_
*M* *_r_* = 489.36
Orthorhombic, 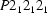

*a* = 10.975 (2) Å
*b* = 12.255 (3) Å
*c* = 17.741 (4) Å
*V* = 2386.2 (8) Å^3^
*Z* = 4
Mo *K*α radiation
μ = 0.31 mm^−1^
*T* = 296 K
0.38 × 0.27 × 0.16 mm

#### Data collection

Bruker APEXII CCD diffractometer
Absorption correction: multi-scan (*SADABS*; Bruker, 2007 ▶) *T*_min_ = 0.892, *T*_max_ = 0.952
12018 measured reflections
4252 independent reflections
2217 reflections with *I* > 2σ(*I*)
*R*_int_ = 0.067

#### Refinement

*R*[*F* ^2^ > 2σ(*F* ^2^)] = 0.053
*wR*(*F* ^2^) = 0.131
*S* = 1.02
4252 reflections
297 parameters
H-atom parameters constrained
Δρ_max_ = 0.24 e Å^−3^
Δρ_min_ = −0.16 e Å^−3^
Absolute structure: Flack (1983 ▶), 1834 Friedel pairs
Flack parameter: 0.01 (10)

### 

Data collection: *APEX2* (Bruker, 2007 ▶); cell refinement: *SAINT* (Bruker, 2007 ▶); data reduction: *SAINT*; program(s) used to solve structure: *SHELXS97* (Sheldrick, 2008 ▶); program(s) used to refine structure: *SHELXL97* (Sheldrick, 2008 ▶); molecular graphics: *ORTEPIII* (Burnett & Johnson, 1996 ▶) and *ORTEP-3 for Windows* (Farrugia, 1997 ▶); software used to prepare material for publication: *SHELXTL* (Sheldrick, 2008 ▶) and *PLATON* (Spek, 2009 ▶).

## Supplementary Material

Crystal structure: contains datablocks I, global. DOI: 10.1107/S1600536811010270/dn2666sup1.cif

Structure factors: contains datablocks I. DOI: 10.1107/S1600536811010270/dn2666Isup2.hkl

Additional supplementary materials:  crystallographic information; 3D view; checkCIF report

## Figures and Tables

**Table 1:** Hydrogen-bond geometry (Å, °) *Cg*3 is the centroid of the C13–C18 ring.

*D*—H⋯*A*	*D*—H	H⋯*A*	*D*⋯*A*	*D*—H⋯*A*
C6—H6B⋯O3^i^	0.96	2.54	3.498 (8)	173
C11—H11A⋯O1^ii^	0.97	2.36	3.281 (6)	159
C21—H21B⋯O2^iii^	0.96	2.44	3.403 (6)	178
C4—H4A⋯*Cg*3^i^	0.96	2.86	3.619 (7)	136

Symmetry codes: (i) 
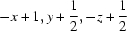
; (ii) 
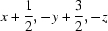
; (iii) 
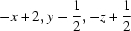
.

**Fig. 1.**
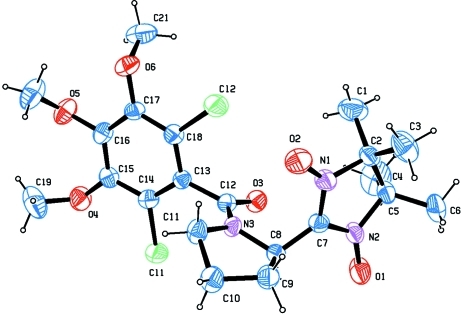
Molecular structure of the title compound (I), showing the atom labeling scheme. Displacement ellipsoids are drawn at the 30% probability level. H atoms are represented as small spheres of arbitrary radii.

## supplementary crystallographic information

### Comment

Nitronyl nitroxides, stable organic radicals, synthesized more than 30 years ago (Osiecki *et al.*, 1968), have received considerable attention recently because of their capability of magnetism, anticancer, antiradiation and antioxidation in biological chemistry and magnetic material fields (Shemsi *et al.*, 2007; Wu *et al.*, 2006). Chiral nitroxides are well chosen as potential precursors of chiralmolecule-based magnets. However, chiral nitronyl nitroxide radicals with the chiral centers sitting very close to the oxyl group are relatively few in the literature (Minguet *et al.*, 2001; Shimono *et al.*, 2004; Tian *et al.*, 2011).

In the title compound, the nitronyl nitroxide ring displays half-chair conformation with puckering parameters, Q2 = 0.096 (5)Å and φ = 272 (3)° (Cremer & Pople, 1975) whereas the pyrrolidine ring has an envelope conformation with puckering parameters Q(2)= 0.393 (6) Å and φ = 104.3 (7)°. The N3—C12—C13—C14, -97.7 (6)°, and N3—C12—C13—C18, 86.3 (6)°, torsion angles the involving the ketone bridging group show that the phenyl and the pyrrolidine rings are nearly perpendicular (Fig. 1). The bond distances and bond angles within the molecule agree with values reported in the Cambridge Structural Database (Allen, 2002).

Intermolecular C—H···O and C—H···π hydrogen bonds stabilize the packing building up a three dimensionnal network (Table 1).

.

### Experimental

2,6-dichloro-3,4,5-trimethoxybenzoylchloride (2.98 g, 10.0 mmol), Et_3_N (3.1 ml) were added to in dry CH_2_Cl_2_ with vigorous stirring in an ice bath. To this mixture, a solution of prolinol (1.0 g, 10 mmol) in dry CH_2_Cl_2_was added dropwise over a period of 20 min. The mixture was warmed to room temperature and stirred for 2 h. Then the reaction mixture was treated with water and extracted with CH_2_Cl_2_. The organic layer wasdried over anhydrous MgSO_4_ and concentrated. The crude product was purified by column chromatography on silica gel using ethyl acetate/petroleum ether (3:1) as eluant, giving a colorless oil product (3.1 g, 83.8%). To a reaction mixture of this product (3.61 g, 10 mmol), TEMPO (0.025 g, 0.16 mmol) and trichloroisocyanuric acid (TCCA, 3.7 g, 16 mmol), CH_2_Cl_2_ was added. Then the mixture was stirred for 20 min and filtered on Celite. The precipitate was purified by column chromatography on silica gel using ethyl acetate/petroleumether/triethylamine (2:1:0.1) as eluant, giving the product 1-(2,6-dichloro-3,4,5-trimethoxybenzoyl)pyrrolidine-2-carbaldehyde (3.10 g, 85.0%). 2,3-Dimethyl-2,3-bis(hydroxylamino) butane (0.74 g, 10.0 mmol) and 1-(2,6-dichloro-3,4,5- trime thoxybenzoyl) pyrrolidine-2-carbaldehyde (1.81 g, 5.0 mmol) were dissolved in methanol. The reaction was stirred for 10 h at reflux temperature, then cooled to room temperature and filtered. The cake was suspended in CH_2_Cl_2_ (150.0 ml) and cooled at ice bath for 10 min. Then the reaction mixture was added to an aqueous solution of NaIO_4_ stirring for 15 min. The aqueous phase was extracted with CH_2_Cl_2_ and the organic layer was combined and dried over MgSO_4_. Then the solvent wasremoved to give a amaranthine residue which was purified by a flash columnchromatography with the elution of n-hexane/ethyl acetate (1:1) to yield thetitle compound (I) as a dark amaranthine powder. Single crystals of compound (I) were obtained from the 1/1 mixed solution of *n*-heptane and dichloromethane.

### Refinement

All H atoms attached to C atoms were fixed geometrically and treated as riding with C—H = 0.96 Å (methyl), 0.97 Å (methylene) and 0.93 Å (aromatic) with *U*_iso_(H) = 1.2*U*_eq_(C) or *U*_iso_(H) = 1.5*U*_eq_(Cmethyl).

### Crystal data

**Table:**

C_21_H_28_Cl_2_N_3_O_6_	*F*(000) = 1028
*M**_r_* = 489.36	*D*_x_ = 1.362 Mg m^−^^3^
Orthorhombic, *P*2_1_2_1_2_1_	Mo *K*α radiation, λ = 0.71073 Å
Hall symbol: P 2ac 2ab	Cell parameters from 1127 reflections
*a* = 10.975 (2) Å	θ = 2.3–16.9°
*b* = 12.255 (3) Å	µ = 0.31 mm^−^^1^
*c* = 17.741 (4) Å	*T* = 296 K
*V* = 2386.2 (8) Å^3^	Block, blue
*Z* = 4	0.38 × 0.27 × 0.16 mm

### Data collection

**Table:**

Bruker APEXII CCD diffractometer	4252 independent reflections
Radiation source: fine-focus sealed tube	2217 reflections with *I* > 2σ(*I*)
graphite	*R*_int_ = 0.067
φ and ω scans	θ_max_ = 25.1°, θ_min_ = 2.0°
Absorption correction: multi-scan (*SADABS*; Bruker, 2007)	*h* = −13→12
*T*_min_ = 0.892, *T*_max_ = 0.952	*k* = −12→14
12018 measured reflections	*l* = −18→21

### Refinement

**Table:**

Refinement on *F*^2^	Hydrogen site location: inferred from neighbouring sites
Least-squares matrix: full	H-atom parameters constrained
*R*[*F*^2^ > 2σ(*F*^2^)] = 0.053	*w* = 1/[σ^2^(*F*_o_^2^) + (0.040*P*)^2^ + 0.1713*P*] where *P* = (*F*_o_^2^ + 2*F*_c_^2^)/3
*wR*(*F*^2^) = 0.131	(Δ/σ)_max_ < 0.001
*S* = 1.02	Δρ_max_ = 0.24 e Å^−^^3^
4252 reflections	Δρ_min_ = −0.16 e Å^−^^3^
297 parameters	Extinction correction: *SHELXL97* (Sheldrick, 2008), Fc^*^=kFc[1+0.001xFc^2^λ^3^/sin(2θ)]^-1/4^
0 restraints	Extinction coefficient: 0.0025 (7)
Primary atom site location: structure-invariant direct methods	Absolute structure: Flack (1983), 1834 Friedel pairs
Secondary atom site location: difference Fourier map	Flack parameter: 0.01 (10)

### Special details

**Table:**

Geometry. All e.s.d.'s (except the e.s.d. in the dihedral angle between two l.s. planes) are estimated using the full covariance matrix. The cell e.s.d.'s are taken into account individually in the estimation of e.s.d.'s in distances, angles and torsion angles; correlations between e.s.d.'s in cell parameters are only used when they are defined by crystal symmetry. An approximate (isotropic) treatment of cell e.s.d.'s is used for estimating e.s.d.'s involving l.s. planes.
Refinement. Refinement of *F*^2^ against ALL reflections. The weighted *R*-factor *wR* and goodness of fit *S* are based on *F*^2^, conventional *R*-factors *R* are based on *F*, with *F* set to zero for negative *F*^2^. The threshold expression of *F*^2^ > σ(*F*^2^) is used only for calculating *R*-factors(gt) *etc*. and is not relevant to the choice of reflections for refinement. *R*-factors based on *F*^2^ are statistically about twice as large as those based on *F*, and *R*- factors based on ALL data will be even larger.

### Fractional atomic coordinates and isotropic or equivalent isotropic displacement parameters (Å^2^)

**Table:**

	*x*	*y*	*z*	*U*_iso_*/*U*_eq_	
Cl1	0.54734 (13)	0.50531 (12)	−0.00306 (8)	0.0831 (5)	
Cl2	0.85841 (13)	0.71203 (11)	0.18279 (8)	0.0843 (5)	
N1	0.6828 (4)	0.9676 (3)	0.1322 (2)	0.0620 (11)	
N2	0.4878 (4)	0.9819 (3)	0.1078 (2)	0.0610 (11)	
N3	0.6736 (3)	0.7723 (3)	0.03080 (19)	0.0511 (10)	
O1	0.3875 (3)	0.9782 (3)	0.0717 (2)	0.0963 (13)	
O2	0.7966 (3)	0.9505 (3)	0.1213 (2)	0.0897 (12)	
O3	0.5442 (3)	0.7190 (3)	0.12213 (17)	0.0648 (9)	
O4	0.6903 (4)	0.3135 (3)	0.0339 (2)	0.0835 (11)	
O5	0.8841 (4)	0.3034 (3)	0.1329 (2)	0.0840 (11)	
O6	0.9682 (3)	0.4954 (3)	0.2006 (2)	0.0741 (10)	
C1	0.6747 (6)	0.9565 (5)	0.2688 (3)	0.106 (2)	
H1A	0.6584	0.8809	0.2591	0.160*	
H1B	0.6307	0.9794	0.3128	0.160*	
H1C	0.7605	0.9666	0.2769	0.160*	
C2	0.6345 (5)	1.0242 (4)	0.2016 (2)	0.0634 (13)	
C3	0.6941 (6)	1.1358 (4)	0.2038 (3)	0.104 (2)	
H3A	0.7810	1.1274	0.2056	0.157*	
H3B	0.6670	1.1745	0.2477	0.157*	
H3C	0.6719	1.1760	0.1594	0.157*	
C4	0.4234 (6)	0.9421 (6)	0.2350 (4)	0.137 (3)	
H4A	0.4187	0.9680	0.2860	0.205*	
H4B	0.4643	0.8728	0.2341	0.205*	
H4C	0.3427	0.9339	0.2149	0.205*	
C5	0.4950 (5)	1.0246 (4)	0.1868 (3)	0.0706 (15)	
C6	0.4332 (7)	1.1325 (5)	0.1905 (4)	0.129 (3)	
H6A	0.4699	1.1812	0.1547	0.194*	
H6B	0.4416	1.1621	0.2403	0.194*	
H6C	0.3484	1.1237	0.1788	0.194*	
C7	0.5945 (5)	0.9425 (4)	0.0838 (3)	0.0547 (12)	
C8	0.6088 (4)	0.8744 (3)	0.0148 (2)	0.0523 (12)	
H8	0.5279	0.8571	−0.0053	0.063*	
C9	0.6861 (5)	0.9281 (4)	−0.0482 (3)	0.0736 (15)	
H9A	0.6349	0.9666	−0.0840	0.088*	
H9B	0.7451	0.9786	−0.0273	0.088*	
C10	0.7487 (5)	0.8315 (5)	−0.0850 (3)	0.0785 (17)	
H10A	0.6946	0.7946	−0.1199	0.094*	
H10B	0.8218	0.8540	−0.1116	0.094*	
C11	0.7795 (4)	0.7592 (4)	−0.0183 (3)	0.0674 (15)	
H11A	0.7893	0.6838	−0.0338	0.081*	
H11B	0.8534	0.7835	0.0065	0.081*	
C12	0.6345 (4)	0.7020 (4)	0.0830 (3)	0.0527 (12)	
C13	0.7062 (4)	0.5988 (4)	0.0917 (3)	0.0496 (11)	
C14	0.6708 (4)	0.5020 (4)	0.0577 (2)	0.0594 (12)	
C15	0.7333 (5)	0.4047 (4)	0.0686 (3)	0.0593 (14)	
C16	0.8309 (5)	0.4042 (4)	0.1181 (3)	0.0604 (13)	
C17	0.8696 (4)	0.4979 (4)	0.1537 (2)	0.0546 (11)	
C18	0.8091 (5)	0.5945 (4)	0.1389 (3)	0.0565 (12)	
C19	0.7769 (6)	0.2553 (6)	−0.0120 (5)	0.133 (3)	
H19A	0.8400	0.3044	−0.0280	0.199*	
H19B	0.7363	0.2259	−0.0554	0.199*	
H19C	0.8121	0.1970	0.0168	0.199*	
C20	1.0077 (5)	0.2909 (5)	0.1148 (4)	0.115 (2)	
H20A	1.0224	0.3193	0.0652	0.172*	
H20B	1.0287	0.2149	0.1160	0.172*	
H20C	1.0565	0.3299	0.1507	0.172*	
C21	0.9414 (5)	0.4668 (5)	0.2767 (3)	0.106 (2)	
H21A	0.8876	0.5202	0.2981	0.159*	
H21B	1.0156	0.4644	0.3053	0.159*	
H21C	0.9030	0.3964	0.2779	0.159*	

### Atomic displacement parameters (Å^2^)

**Table:**

	*U*^11^	*U*^22^	*U*^33^	*U*^12^	*U*^13^	*U*^23^
Cl1	0.0821 (9)	0.0807 (9)	0.0865 (10)	0.0042 (8)	−0.0286 (8)	−0.0133 (8)
Cl2	0.0864 (11)	0.0714 (9)	0.0951 (10)	−0.0046 (8)	−0.0274 (9)	−0.0111 (7)
N1	0.049 (3)	0.071 (3)	0.066 (3)	−0.003 (2)	−0.003 (2)	−0.011 (2)
N2	0.049 (3)	0.062 (3)	0.072 (3)	0.003 (2)	−0.006 (2)	−0.024 (2)
N3	0.047 (2)	0.055 (2)	0.052 (2)	0.009 (2)	0.0078 (19)	0.0005 (19)
O1	0.056 (2)	0.103 (3)	0.130 (3)	0.015 (2)	−0.017 (2)	−0.052 (2)
O2	0.054 (2)	0.110 (3)	0.105 (3)	0.001 (2)	−0.014 (2)	−0.015 (2)
O3	0.056 (2)	0.074 (2)	0.065 (2)	0.0052 (19)	0.0137 (19)	0.0060 (18)
O4	0.088 (3)	0.059 (2)	0.103 (3)	−0.005 (2)	−0.010 (2)	−0.013 (2)
O5	0.073 (3)	0.061 (2)	0.118 (3)	0.007 (2)	0.001 (2)	0.018 (2)
O6	0.053 (2)	0.090 (2)	0.079 (3)	0.006 (2)	−0.0084 (19)	0.012 (2)
C1	0.141 (6)	0.108 (5)	0.070 (4)	−0.009 (4)	−0.021 (4)	0.014 (3)
C2	0.085 (4)	0.053 (3)	0.053 (3)	−0.005 (3)	−0.006 (3)	−0.007 (2)
C3	0.145 (6)	0.073 (4)	0.095 (5)	−0.035 (4)	0.014 (4)	−0.013 (3)
C4	0.110 (6)	0.158 (7)	0.142 (6)	−0.001 (5)	0.067 (5)	0.019 (5)
C5	0.089 (4)	0.061 (4)	0.062 (3)	0.010 (3)	0.003 (3)	−0.015 (3)
C6	0.146 (6)	0.113 (5)	0.129 (6)	0.061 (5)	−0.032 (5)	−0.064 (5)
C7	0.050 (3)	0.060 (3)	0.054 (3)	−0.002 (3)	−0.004 (3)	−0.001 (2)
C8	0.051 (3)	0.058 (3)	0.048 (3)	0.004 (2)	−0.004 (2)	−0.006 (2)
C9	0.092 (4)	0.067 (4)	0.062 (3)	0.013 (3)	0.009 (3)	0.006 (3)
C10	0.083 (4)	0.087 (4)	0.065 (4)	0.014 (3)	0.029 (3)	0.011 (3)
C11	0.071 (4)	0.065 (4)	0.066 (4)	0.011 (3)	0.021 (3)	0.007 (3)
C12	0.053 (3)	0.055 (3)	0.050 (3)	0.001 (3)	−0.002 (3)	0.000 (2)
C13	0.045 (3)	0.053 (3)	0.051 (3)	0.002 (2)	0.003 (2)	0.001 (2)
C14	0.056 (3)	0.067 (4)	0.056 (3)	−0.002 (3)	−0.002 (2)	0.003 (3)
C15	0.059 (4)	0.054 (3)	0.065 (3)	−0.010 (3)	0.002 (3)	−0.005 (3)
C16	0.062 (4)	0.049 (3)	0.071 (3)	0.001 (3)	0.005 (3)	0.011 (3)
C17	0.050 (3)	0.055 (3)	0.059 (3)	−0.004 (3)	−0.008 (2)	0.004 (3)
C18	0.061 (3)	0.051 (3)	0.057 (3)	−0.004 (3)	0.001 (3)	0.002 (2)
C19	0.142 (6)	0.124 (6)	0.132 (6)	−0.007 (5)	0.017 (5)	−0.062 (5)
C20	0.070 (4)	0.094 (5)	0.180 (7)	0.017 (4)	−0.001 (4)	0.004 (5)
C21	0.083 (4)	0.157 (6)	0.078 (4)	0.005 (4)	−0.019 (4)	0.026 (4)

### Geometric parameters (Å, °)

**Table:**

Cl1—C14	1.731 (5)	C5—C6	1.487 (7)
Cl2—C18	1.724 (5)	C6—H6A	0.9600
N1—O2	1.281 (5)	C6—H6B	0.9600
N1—C7	1.331 (5)	C6—H6C	0.9600
N1—C2	1.510 (6)	C7—C8	1.490 (6)
N2—O1	1.274 (4)	C8—C9	1.550 (6)
N2—C7	1.336 (5)	C8—H8	0.9800
N2—C5	1.499 (6)	C9—C10	1.516 (6)
N3—C12	1.335 (5)	C9—H9A	0.9700
N3—C11	1.461 (5)	C9—H9B	0.9700
N3—C8	1.468 (5)	C10—C11	1.516 (6)
O3—C12	1.228 (5)	C10—H10A	0.9700
O4—C15	1.359 (5)	C10—H10B	0.9700
O4—C19	1.441 (7)	C11—H11A	0.9700
O5—C16	1.391 (5)	C11—H11B	0.9700
O5—C20	1.403 (6)	C12—C13	1.497 (6)
O6—C17	1.366 (5)	C13—C14	1.387 (6)
O6—C21	1.425 (6)	C13—C18	1.407 (6)
C1—C2	1.517 (6)	C14—C15	1.390 (6)
C1—H1A	0.9600	C15—C16	1.386 (6)
C1—H1B	0.9600	C16—C17	1.377 (6)
C1—H1C	0.9600	C17—C18	1.383 (6)
C2—C3	1.516 (6)	C19—H19A	0.9600
C2—C5	1.554 (7)	C19—H19B	0.9600
C3—H3A	0.9600	C19—H19C	0.9600
C3—H3B	0.9600	C20—H20A	0.9600
C3—H3C	0.9600	C20—H20B	0.9600
C4—C5	1.539 (7)	C20—H20C	0.9600
C4—H4A	0.9600	C21—H21A	0.9600
C4—H4B	0.9600	C21—H21B	0.9600
C4—H4C	0.9600	C21—H21C	0.9600
			
O2—N1—C7	125.1 (4)	C10—C9—C8	103.1 (4)
O2—N1—C2	122.7 (4)	C10—C9—H9A	111.1
C7—N1—C2	112.1 (4)	C8—C9—H9A	111.1
O1—N2—C7	125.7 (4)	C10—C9—H9B	111.1
O1—N2—C5	121.9 (4)	C8—C9—H9B	111.1
C7—N2—C5	112.2 (4)	H9A—C9—H9B	109.1
C12—N3—C11	126.8 (4)	C11—C10—C9	102.8 (4)
C12—N3—C8	121.9 (4)	C11—C10—H10A	111.2
C11—N3—C8	111.3 (3)	C9—C10—H10A	111.2
C15—O4—C19	115.7 (5)	C11—C10—H10B	111.2
C16—O5—C20	117.4 (4)	C9—C10—H10B	111.2
C17—O6—C21	114.8 (4)	H10A—C10—H10B	109.1
C2—C1—H1A	109.5	N3—C11—C10	103.0 (4)
C2—C1—H1B	109.5	N3—C11—H11A	111.2
H1A—C1—H1B	109.5	C10—C11—H11A	111.2
C2—C1—H1C	109.5	N3—C11—H11B	111.2
H1A—C1—H1C	109.5	C10—C11—H11B	111.2
H1B—C1—H1C	109.5	H11A—C11—H11B	109.1
N1—C2—C1	106.7 (4)	O3—C12—N3	122.9 (4)
N1—C2—C3	106.5 (4)	O3—C12—C13	120.6 (4)
C1—C2—C3	110.3 (4)	N3—C12—C13	116.6 (4)
N1—C2—C5	102.1 (4)	C14—C13—C18	116.9 (4)
C1—C2—C5	114.9 (4)	C14—C13—C12	122.0 (4)
C3—C2—C5	115.3 (4)	C18—C13—C12	121.0 (4)
C2—C3—H3A	109.5	C13—C14—C15	122.4 (4)
C2—C3—H3B	109.5	C13—C14—Cl1	118.0 (4)
H3A—C3—H3B	109.5	C15—C14—Cl1	119.6 (4)
C2—C3—H3C	109.5	O4—C15—C16	123.5 (5)
H3A—C3—H3C	109.5	O4—C15—C14	118.1 (5)
H3B—C3—H3C	109.5	C16—C15—C14	118.3 (4)
C5—C4—H4A	109.5	C17—C16—C15	121.7 (5)
C5—C4—H4B	109.5	C17—C16—O5	121.6 (5)
H4A—C4—H4B	109.5	C15—C16—O5	116.6 (4)
C5—C4—H4C	109.5	O6—C17—C16	120.3 (5)
H4A—C4—H4C	109.5	O6—C17—C18	121.0 (4)
H4B—C4—H4C	109.5	C16—C17—C18	118.6 (4)
C6—C5—N2	109.1 (4)	C17—C18—C13	122.0 (4)
C6—C5—C4	109.1 (5)	C17—C18—Cl2	118.6 (4)
N2—C5—C4	105.3 (5)	C13—C18—Cl2	119.3 (4)
C6—C5—C2	116.4 (5)	O4—C19—H19A	109.5
N2—C5—C2	102.1 (4)	O4—C19—H19B	109.5
C4—C5—C2	114.0 (5)	H19A—C19—H19B	109.5
C5—C6—H6A	109.5	O4—C19—H19C	109.5
C5—C6—H6B	109.5	H19A—C19—H19C	109.5
H6A—C6—H6B	109.5	H19B—C19—H19C	109.5
C5—C6—H6C	109.5	O5—C20—H20A	109.5
H6A—C6—H6C	109.5	O5—C20—H20B	109.5
H6B—C6—H6C	109.5	H20A—C20—H20B	109.5
N1—C7—N2	110.5 (4)	O5—C20—H20C	109.5
N1—C7—C8	125.6 (4)	H20A—C20—H20C	109.5
N2—C7—C8	123.8 (4)	H20B—C20—H20C	109.5
N3—C8—C7	111.7 (4)	O6—C21—H21A	109.5
N3—C8—C9	103.6 (4)	O6—C21—H21B	109.5
C7—C8—C9	114.4 (4)	H21A—C21—H21B	109.5
N3—C8—H8	109.0	O6—C21—H21C	109.5
C7—C8—H8	109.0	H21A—C21—H21C	109.5
C9—C8—H8	109.0	H21B—C21—H21C	109.5
			
O2—N1—C2—C1	−59.4 (5)	C8—N3—C11—C10	−22.5 (5)
C7—N1—C2—C1	123.1 (5)	C9—C10—C11—N3	37.7 (5)
O2—N1—C2—C3	58.4 (6)	C11—N3—C12—O3	−179.9 (4)
C7—N1—C2—C3	−119.1 (5)	C8—N3—C12—O3	−1.8 (7)
O2—N1—C2—C5	179.6 (4)	C11—N3—C12—C13	−0.4 (6)
C7—N1—C2—C5	2.2 (5)	C8—N3—C12—C13	177.7 (4)
O1—N2—C5—C6	−50.3 (7)	O3—C12—C13—C14	81.7 (6)
C7—N2—C5—C6	134.2 (5)	N3—C12—C13—C14	−97.8 (5)
O1—N2—C5—C4	66.7 (6)	O3—C12—C13—C18	−94.3 (6)
C7—N2—C5—C4	−108.8 (5)	N3—C12—C13—C18	86.2 (5)
O1—N2—C5—C2	−173.9 (4)	C18—C13—C14—C15	−0.5 (7)
C7—N2—C5—C2	10.5 (5)	C12—C13—C14—C15	−176.7 (4)
N1—C2—C5—C6	−125.7 (5)	C18—C13—C14—Cl1	−178.0 (3)
C1—C2—C5—C6	119.3 (5)	C12—C13—C14—Cl1	5.8 (6)
C3—C2—C5—C6	−10.7 (7)	C19—O4—C15—C16	−58.5 (7)
N1—C2—C5—N2	−7.0 (5)	C19—O4—C15—C14	126.1 (6)
C1—C2—C5—N2	−122.1 (4)	C13—C14—C15—O4	178.9 (4)
C3—C2—C5—N2	107.9 (5)	Cl1—C14—C15—O4	−3.6 (6)
N1—C2—C5—C4	105.9 (5)	C13—C14—C15—C16	3.1 (7)
C1—C2—C5—C4	−9.2 (6)	Cl1—C14—C15—C16	−179.3 (4)
C3—C2—C5—C4	−139.1 (5)	O4—C15—C16—C17	−178.3 (4)
O2—N1—C7—N2	−172.9 (5)	C14—C15—C16—C17	−2.8 (7)
C2—N1—C7—N2	4.5 (5)	O4—C15—C16—O5	−1.3 (7)
O2—N1—C7—C8	10.8 (7)	C14—C15—C16—O5	174.2 (4)
C2—N1—C7—C8	−171.8 (4)	C20—O5—C16—C17	−63.0 (7)
O1—N2—C7—N1	174.9 (5)	C20—O5—C16—C15	120.0 (5)
C5—N2—C7—N1	−9.8 (5)	C21—O6—C17—C16	−86.5 (6)
O1—N2—C7—C8	−8.7 (8)	C21—O6—C17—C18	95.5 (5)
C5—N2—C7—C8	166.6 (4)	C15—C16—C17—O6	−178.2 (4)
C12—N3—C8—C7	56.3 (5)	O5—C16—C17—O6	4.9 (7)
C11—N3—C8—C7	−125.3 (4)	C15—C16—C17—C18	−0.2 (7)
C12—N3—C8—C9	179.9 (4)	O5—C16—C17—C18	−177.1 (4)
C11—N3—C8—C9	−1.7 (5)	O6—C17—C18—C13	−178.9 (4)
N1—C7—C8—N3	48.9 (6)	C16—C17—C18—C13	3.1 (7)
N2—C7—C8—N3	−127.0 (5)	O6—C17—C18—Cl2	−1.5 (6)
N1—C7—C8—C9	−68.5 (6)	C16—C17—C18—Cl2	−179.5 (4)
N2—C7—C8—C9	115.6 (5)	C14—C13—C18—C17	−2.7 (7)
N3—C8—C9—C10	25.2 (5)	C12—C13—C18—C17	173.5 (4)
C7—C8—C9—C10	147.1 (4)	C14—C13—C18—Cl2	179.8 (3)
C8—C9—C10—C11	−38.9 (5)	C12—C13—C18—Cl2	−3.9 (6)
C12—N3—C11—C10	155.8 (4)		

### Hydrogen-bond geometry (Å, °)

**Table:**

Cg3 is the centroid of the C13–C18 ring.

**Table:**

*D*—H···*A*	*D*—H	H···*A*	*D*···*A*	*D*—H···*A*
C6—H6B···O3^i^	0.96	2.54	3.498 (8)	173
C11—H11A···O1^ii^	0.97	2.36	3.281 (6)	159
C21—H21B···O2^iii^	0.96	2.44	3.403 (6)	178
C4—H4A···Cg3^i^	0.96	2.86	3.619 (7)	136

Symmetry codes: (i) −*x*+1, *y*+1/2, −*z*+1/2; (ii) *x*+1/2, −*y*+3/2, −*z*; (iii) −*x*+2, *y*−1/2, −*z*+1/2.
